# Trends in menopausal hormone therapy use among postmenopausal women in South Korea: analysis of KNHANES 2005–2024

**DOI:** 10.3389/fpubh.2026.1847250

**Published:** 2026-06-17

**Authors:** Kisok Kim, Hyejin Park

**Affiliations:** 1 College of Pharmacy, Keimyung University, Daegu, Republic of Korea; 2 Department of Health Sciences, Dongduk Women's University, Seoul, Republic of Korea

**Keywords:** KNHANES, Korea, menopausal hormone therapy, postmenopause, trends

## Abstract

Menopausal Hormone Therapy (MHT) has long been used to manage postmenopausal symptoms in Republic of Korea. However, long-term trends in MHT use among postmenopausal Korean women remain incompletely characterized. This study examined temporal trends in the lifetime prevalence of MHT use and sociodemographic correlates using nationally representative data spanning two decades. We conducted a secondary analysis of the Korea National Health and Nutrition Examination Survey (KNHANES) cycles 3 (2005), 4 (2007–2009), 5 (2010–2012), and 9 (2024), including 11,407 postmenopausal women aged ≥40 years. Survey-weighted prevalence estimates with 95% confidence intervals were calculated. Rao-Scott chi-square tests assessed differences across survey cycles and demographic subgroups, while survey-weighted logistic regression models evaluated temporal trends. Overall weighted lifetime prevalence of MHT use was 15.0% (95% CI: 14.1–15.9%), increasing significantly from 13.8% in 2005 to 17.5% in 2024 (*p* for trend = 0.04). MHT use was significantly associated with younger age, higher education, and higher income (all *p* for trend < 0.01). Comparing the past period (2005–2012, prevalence 14.4%) with the recent period (2024, prevalence 17.5%) revealed a significant increase (*p* = 0.01). Age-specific analysis demonstrated substantial increases in older age groups: among women aged ≥70 years, prevalence rose from 3.5 to 12.5% (*p* < 0.01). In addition, prevalence among women with lower education levels (< middle school) significantly increased from 10.1 to 16.0% (*p* < 0.01). These patterns may reflect evolving clinical practice and patient preferences, possibly driven by accumulating evidence supporting individualized MHT use for symptomatic relief in appropriately selected women.

## Introduction

Menopausal Hormone Therapy (MHT) remains the most effective treatment for vasomotor symptoms and genitourinary syndrome of menopause. The publication of the Women's Health Initiative (WHI) randomized controlled trial results in 2002 fundamentally altered the landscape of MHT worldwide (1, 2). The WHI study reported increased risks of cardiovascular events, stroke, venous thromboembolism, and breast cancer with combined estrogen-progestin therapy, leading to the early termination of that trial arm (3). These findings triggered immediate and sustained declines in MHT prescribing across multiple countries, including the United States, Australia, United Kingdom, Spain, and Canada (1, 4–8).

In the United States, national prescription data documented substantial declines in hormone therapy use immediately following the WHI publication, with decreases concentrated in combined oral standard-dose and high-dose preparations (1). Similar patterns emerged internationally: Australian data showed substantial drops in total MHT use after 2002, with oral and medium-strength estrogens declining most sharply while vaginal preparations remained relatively stable (4). In the United Kingdom, MHT use among postmenopausal women declined substantially in the years following the WHI publication (6). Spanish studies documented marked reversals with large decreases in prevalence and new users over 5 years following WHI publication (7).

The global shift in prescribing patterns extended beyond simple reductions in use. Clinicians and patients moved toward lower-dose systemic regimens (including transdermal estradiol formulations, which achieve therapeutic serum estradiol levels and are correctly classified as systemic therapies) and, separately, local vaginal estrogen therapies for genitourinary symptoms, reserving systemic MHT for symptomatic relief rather than chronic disease prevention (2, 9). Subsequent re-analyses and subgroup data from WHI indicated more favorable risk-benefit profiles when MHT is initiated in younger postmenopausal women or used for short durations for symptom control (9). Current evidence supports individualized MHT use, with randomized trial data confirming effectiveness for relieving vasomotor and urogenital symptoms and improving short-term health-related quality of life in symptomatic women (9).

In South Korea, the impact of WHI on MHT patterns has been documented through national pharmacy and sales data. Korean analyses reported post-2002 reductions in systemic hormone sales and parallel shifts toward low-dose preparations and alternative agents such as tibolone (10). However, comprehensive long-term trends in the lifetime prevalence of MHT use among Korean postmenopausal women spanning the post-WHI era through recent years remain poorly understood. Specifically, no prior Korean study has used nationally representative survey data to examine population-level lifetime MHT prevalence extending to 2024, nor to systematically assess sociodemographic disparities—including age, education, and income—across this full period. Understanding these trends is crucial for informing clinical practice, public health policy, and patient counseling. This study aimed to examine trends in the lifetime prevalence of MHT use among postmenopausal Korean women from 2005 to 2024 using nationally representative KNHANES data, and to identify sociodemographic factors associated with MHT use across this period.

## Materials and methods

### Study population

**Figure 1:**
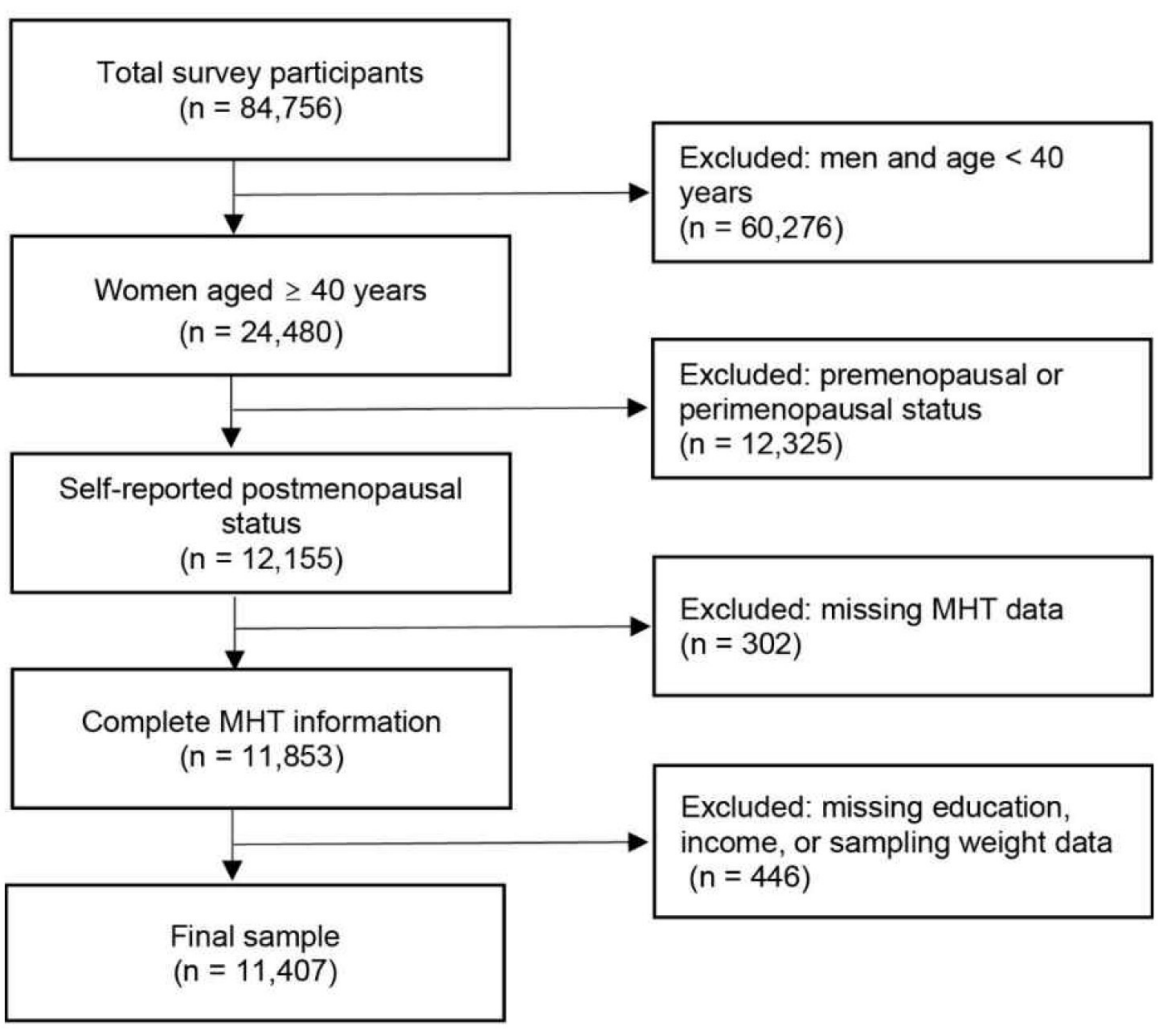
Flow chart of study sample selection. MHT, menopausal hormone therapy.

This study employed a secondary analysis of cross-sectional data from the Korea National Health and Nutrition Examination Survey (KNHANES), a nationally representative survey conducted by the Korea Disease Control and Prevention Agency. KNHANES uses a complex, multistage, stratified probability sampling design to represent the non-institutionalized civilian population of South Korea. We analyzed KNHANES data from cycles 3 (2005), 4 (2007–2009), 5 (2010–2012), and 9 (2024), which include data on MHT use. Cycles 6 (2013–2015), 7 (2016–2018), and 8 (2019–2021) did not collect MHT data and were therefore excluded, resulting in a 12-year gap between Cycle 5 and Cycle 9. The study population comprised postmenopausal women aged 40 years and older who participated in the selected KNHANES cycles. Postmenopausal status was defined based on self-reported cessation of menstruation. Women with missing data on menopausal status or MHT use were excluded from the analysis. The final analytic sample included 11,407 postmenopausal women (Figure 1). The protocol of this study was approved by the Korean Ministry of Health and Welfare, and the research was conducted in accordance with the principles of the Declaration of Helsinki. All study participants provided informed consent.

### Data collection

The primary outcome was self-reported MHT use. Data were obtained from the KNHANES health interview component, which asked: “have you ever taken female hormone medications for at least 1 month due to menopause?” Participants who responded affirmatively were classified as MHT users. Demographic characteristics included age (categorized as 40–59, 60–69, and ≥70 years), education level (< middle school, middle school, > middle school), and household income (quartiles 1–4, with quartile 1 representing the lowest income).

### Statistical analysis

All analyses incorporated KNHANES survey weights to account for the complex sampling design and to produce nationally representative estimates. When multiple KNHANES cycles were pooled, survey-specific weights were harmonized across years and recalibrated by dividing the original annual weights by the total number of pooled survey years, in accordance with KNHANES analytic guidelines for pooled analyses. Weighted prevalence of MHT use and 95% confidence intervals were calculated overall, by survey cycle, and by demographic characteristics. To assess temporal trends across survey cycles, we used survey-weighted logistic regression models treating survey cycle (coded 1–4, corresponding to 2005, 2007–2009, 2010–2012, and 2024) as a continuous variable. Rao-Scott chi-square tests, which adjust for the complex survey design, were used to test for differences in MHT prevalence across categorical variables including survey cycle, age group, education level, and income quartile. For comparison between time periods, we grouped cycles 3, 4, and 5 (2005–2012) as the “past period” and cycle 9 (2024) as the “recent period.” Rao-Scott chi-square tests compared MHT prevalence between these two periods overall and within demographic subgroups. Interaction terms between cycle and demographic variables (age, education, income) were included in logistic regression models to test whether temporal trends differed across subgroups. Statistical significance was set at *p* < 0.05. All analyses were performed using SAS version 9.4 (SAS Institute Inc., Cary, NC, USA), applying survey procedures that accounted for the complex sampling design of KNHANES, including stratification, clustering, and sampling weights.

## Results

Among 11,407 postmenopausal women, the overall weighted lifetime prevalence of MHT use was 15.0% (95% CI: 14.1–15.9%). Table 1 presents lifetime prevalence of MHT use by survey cycle and demographic characteristics. Prevalence varied significantly across survey cycles (*p* for trend = 0.04), increasing from 13.8% (95% CI: 11.7–15.9%) in cycle 3 (2005) to 17.5% (95% CI: 15.3–19.7%) in cycle 9 (2024). MHT use varied significantly by age (*p* for trend < 0.01), with highest prevalence among women aged 40–59 years (19.2%, 95% CI: 17.6–20.9%), declining to 6.5% (95% CI: 5.5–7.5%) among women aged ≥70 years. Education level was strongly associated with MHT use (*p* for trend < 0.01), with prevalence of 10.7% (95% CI: 9.7–11.7%) among women with less than middle school education, 20.3% (95% CI: 17.9–22.7%) among those with middle school education, and 20.8% (95% CI: 19.0–22.7%) among those with greater than middle school education. Similarly, household income showed a significant gradient (*p* for trend < 0.01), with prevalence increasing from 12.2% (95% CI: 10.6–13.9%) in the lowest income quartile to 18.1% (95% CI: 16.2–19.9%) in the highest quartile.

**Table 1:** Number and weighted prevalence (95% CI) of menopausal hormone therapy use by survey cycle and demographic characteristics.

Characteristics	*N*	Weighted prevalence (%) (95% CI)	*P* for trend^a^
Total	11,407	15.0 (14.1–15.9)	
Survey cycle			
Cycle 3 (2005)	1,071	13.8 (11.7–15.9)	0.04
Cycle 4 (2007–2009)	3,596	14.4 (12.6–16.1)	
Cycle 5 (2010–2012)	5,125	14.5 (13.2–15.7)	
Cycle 9 (2024)	1,615	17.5 (15.3–19.7)	
Age (years)			
40–59	3,863	19.2 (17.6–20.9)	<0.01
60–69	3,875	17.5 (15.8–19.1)	
≥70	3,669	6.5 (5.5–7.5)	
Education			
<Middle school	6,977	10.7 (9.7–11.7)	<0.01
Middle school	1,645	20.3 (17.9–22.7)	
>Middle school	2,785	20.8 (19.0–22.7)	
Income			
Quartile 1 (lowest)	2,880	12.2 (10.6–13.9)	<0.01
Quartile 2	2,899	14.6 (13.0–16.3)	
Quartile 3	2,849	15.1 (13.4–16.7)	
Quartile 4 (highest)	2,779	18.1 (16.2–19.9)	

^a^*P* for trend values were calculated by treating each categorical variable as a continuous variable in the survey-weighted logistic regression model.

**Figure 2:**
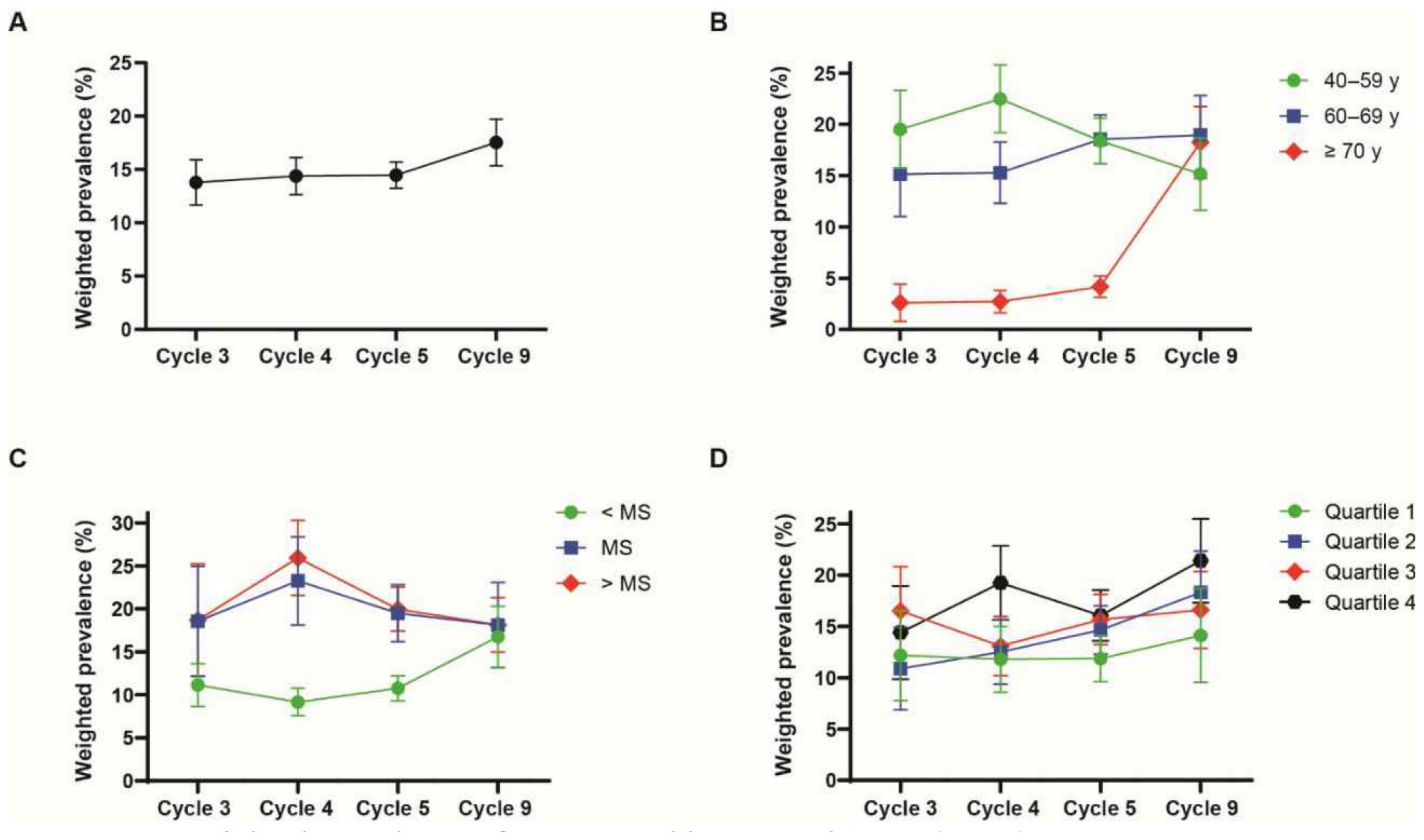
Weighted prevalence of menopausal hormone therapy (MHT) use among postmenopausal Korean women across KNHANES survey cycles and demographic subgroups. **(A)** Overall weighted prevalence of MHT use across four KNHANES survey cycles: Cycle 3 (2005), Cycle 4 (2007–2009), Cycle 5 (2010–2012), and Cycle 9 (2024). Prevalence increased significantly over the study period (*p* for trend = 0.04). **(B)** Age-specific weighted prevalence of MHT use by survey cycle, stratified into three age groups: 40–59 years (green circles), 60–69 years (blue squares), and ≥70 years (red diamonds). A significant interaction between age group and survey cycle was observed (*p* for interaction < 0.001). **(C)** Education-specific weighted prevalence of MHT use by survey cycle, stratified into three groups: < middle school (green circles), middle school (blue squares), and > middle school (red diamonds). Trends differed significantly by education level (*p* for interaction < 0.001). **(D)** Income-specific weighted prevalence of MHT use by survey cycle, stratified into quartiles: quartile 1 (green circles), quartile 2 (blue squares), quartile 3 (red diamonds), and quartile 4 (black circles). No significant interaction between income quartile and survey cycle was observed (*p* for interaction = 0.813). MHT, menopausal hormone therapy; KNHANES, Korea National Health and Nutrition Examination Survey; MS, middle school.

Figure 2 presents the lifetime prevalence of MHT use by survey cycle and demographic characteristics. MHT use showed significantly different temporal trends across survey cycles by age group (*p* for interaction < 0.001), with women aged 40–59 years and 60–69 years showing relatively stable or declining trends, while women aged ≥70 years demonstrated a marked increase over the study period. Trends also varied significantly by education level (*p* for interaction < 0.001). Women with higher education exhibited temporal trends that differed from those observed among women with lower educational attainment. In contrast, MHT use trends did not differ significantly across income quartiles (*p* for interaction = 0.813).

Table 2 presents the weighted lifetime prevalence of MHT use, comparing the past period (2005–2012) with the recent period (2024) across demographic characteristics. The overall lifetime prevalence of MHT use increased significantly from 14.4% (95% CI: 13.4–15.4%) in the past period to 17.5% (95% CI: 15.3–19.7%) in the recent period (*p* = 0.01). Age-specific analyses revealed divergent trends. Among older age groups, MHT prevalence showed a marked and statistically significant increase: in women aged ≥70 years, it rose from 3.5% (95% CI: 2.7–4.3%) to 12.5% (95% CI: 8.0–17.1%) (*p* < 0.01). In women aged 40–59 years, MHT prevalence declined significantly from 19.9% (95% CI: 18.4–21.4%) to 15.1% (95% CI: 12.5–17.7%) (*p* = 0.03). No significant change was observed in women aged 60–69 years [past: 17.1% (95% CI: 15.6–18.6%) vs. recent: 19.0% (95% CI: 16.4–21.6%); *p* = 0.37]. Regarding educational attainment, women with less than a middle school education showed a significant increase in MHT prevalence, from 10.1% (95% CI: 9.0–11.1%) to 16.0% (95% CI: 12.6–19.4%) (*p* < 0.01). However, no significant changes were noted among women with a middle school education or higher. Furthermore, MHT prevalence did not reach statistical significance across any of the income quartiles between the two periods.

**Table 2:** Weighted prevalence (95% CI) of menopausal hormone therapy use between past and recent periods by demographic characteristics.

Characteristics	Past (2005–2012) prevalence (95% CI)	Recent (2024) prevalence (95% CI)	*P* value^a^
Total	14.4 (13.4–15.4)	17.5 (15.3–19.7)	0.01
Age (years)			
40–59	19.9 (18.1–21.7)	15.1 (11.6–18.7)	0.03
60–69	17.1 (15.2–18.9)	19.0 (15.1–22.8)	0.37
≥70	3.5 (2.8–4.3)	12.5 (8.0–17.1)	<0.01
Education			
< Middle school	10.1 (9.0–11.1)	16.0 (12.6–19.4)	<0.01
Middle school	20.8 (18.1–23.5)	18.4 (13.4–23.3)	0.41
> Middle school	22.2 (20.0–24.5)	18.2 (15.0–21.3)	0.05
Income			
Quartile 1 (lowest)	11.8 (10.0–13.6)	14.2 (9.7–18.7)	0.31
Quartile 2	13.9 (12.1–15.8)	18.1 (14.2–22.1)	0.05
Quartile 3	14.8 (13.0–16.6)	16.3 (12.6–19.9)	0.47
Quartile 4 (highest)	17.3 (15.3–19.4)	21.1 (17.1–25.1)	0.09

^a^*P* values were calculated using the Rao-Scott chi-square test to compare prevalence between the two periods.

## Discussion

This nationally representative analysis of 11,407 postmenopausal Korean women showed a significant increasing trend in the lifetime prevalence of MHT use from 2005 to 2024, with overall prevalence rising from 13.8 to 17.5%. The observed trends in Korea present a complex pattern that differs from the immediate post–WHI experience reported in many Western countries. Following the WHI publication in 2002, MHT use declined rapidly and substantially worldwide (9, 11, 12), and similar reductions in systemic hormone use were also documented in Republic of Korea (13, 14). However, our findings from 2005 to 2024 suggest a subsequent recovery and evolution in MHT use patterns. The increase observed by 2024 may, in part, reflect accumulating evidence from WHI re-analyses and subsequent studies indicating more favorable risk–benefit profiles when MHT is initiated in younger postmenopausal women or used for appropriate durations for symptom control (15–17).

A notable finding of this study is the divergent age-specific trend in MHT use. Lifetime prevalence increased markedly among older women (≥70 years: 3.5 to 12.5%), while significantly declining among younger women aged 40–59 years (19.9–15.1%) and remaining relatively stable among those aged 60–69 years. Several mechanisms may underlie this finding and should be interpreted with caution. First, older MHT users in 2024 may reflect long-term continuation rather than new initiation at older ages; women who initiated MHT in their 50's during the 2000's or early 2010's may have continued treatment and subsequently transitioned into older age groups by 2024. This interpretation aligns with prior evidence indicating that some women continued MHT use because of perceived benefits in symptom control despite the findings of the WHI studies (18, 19). Second, cohort effects may contribute, as older women surveyed in 2024 belong to birth cohorts with different healthcare experiences and attitudes toward MHT compared with those surveyed in 2005. Third, survivor bias may contribute, as women who tolerated MHT well may have been more likely to remain represented in the survey population. Fourth, evolving clinical guidelines and the “timing hypothesis” may have supported prolonged use or re-initiation of MHT among selected older women. However, because our cross-sectional design and lifetime prevalence measure cannot distinguish among these mechanisms, all interpretations remain speculative. In contrast, the decline among younger women may reflect changing symptom perceptions, increased use of non-hormonal alternatives, or persistent concerns regarding MHT-related risks despite accumulating evidence supporting its safety and efficacy when appropriately prescribed (15–17).

MHT use in this study was consistently associated with higher education and income levels. These findings align with international evidence demonstrating that higher socioeconomic status is associated with greater MHT use (20, 21). Such disparities may reflect differences in healthcare access, health literacy, treatment-seeking behavior, and prescribing practices. Notably, the socioeconomic gradient in MHT use appeared to narrow in the more recent period (2024) compared with earlier years (2005–2012). This convergence may indicate improved access to menopausal care across socioeconomic groups, evolving prescribing practices, or changing patient preferences and risk perceptions.

Several limitations should be considered when interpreting these findings. First, the cross-sectional design precludes assessment of individual-level longitudinal changes in MHT use. Furthermore, the survey question captures lifetime prevalence (ever-use for at least 1 month) rather than current use, which prevents us from distinguishing between current users, past users who discontinued, long-term continuers, and new initiators; this limits interpretation of observed trends in terms of current treatment patterns or guideline adherence. As such, we cannot distinguish between long-term continuation, new initiation, or intermittent use. Longitudinal studies are needed to better characterize individual treatment trajectories. Second, the data do not differentiate between MHT formulations (systemic vs. local), dosing regimens, or hormone types (e.g., estrogen-only vs. combined estrogen–progestin; oral vs. transdermal). This is important, as prior studies have demonstrated that risks and benefits vary by formulation (22–24), and international trends indicate shifts toward vaginal, transdermal, and lower-dose therapies (12, 23, 24). Third, information on clinical indications, symptom severity, duration of therapy, hysterectomy status, and timing since menopause onset was unavailable. These variables are essential for assessing guideline concordance and risk-benefit profiles; their absence substantially limits the clinical interpretability of our findings and prevents assessment of whether MHT use was appropriately targeted or consistent with current clinical guidelines. Fourth, self-reported MHT use may be subject to recall bias: women may inaccurately recall whether they used hormone therapy, particularly if use occurred many years prior to the survey. This bias may vary by age (older women recalling more distant use) or education level, potentially introducing systematic error into prevalence estimates. Although standardized KNHANES procedures likely minimized such errors, recall bias cannot be entirely excluded. Fifth, our analysis spans nearly two decades, during which healthcare systems, prescribing behaviors, and patient populations have evolved. Unmeasured temporal changes in these factors may have influenced the observed trends. Sixth, the survey question did not provide participants with an explicit definition of “female hormone medications”; therefore, responses may reflect varying interpretations, and potential misclassification (e.g., inconsistent inclusion of vaginal estrogen preparations or tibolone) should be considered when interpreting the results. Seventh, no adjustment for multiple testing (e.g., Bonferroni correction) was applied to subgroup analyses. Given the exploratory nature of these analyses, findings should be interpreted as hypothesis-generating, and some statistically significant associations may represent Type I errors. Finally, treating survey cycle as a continuous variable assumes a linear temporal trend, which may not fully capture non-linear patterns; the 12-year data gap between Cycles 5 and 9 also limits characterization of trends during 2013–2023.

The Korean experience appears to differ from the sustained low MHT utilization documented in many Western countries following the initial WHI-driven decline, highlighting the importance of understanding country-specific factors that influence menopausal care. Future research should examine formulation-specific trends, indications for use, duration of therapy, and clinical outcomes to better characterize the appropriateness and safety of evolving MHT prescribing patterns in Korea. Longitudinal studies tracking individual-level changes in MHT use and their determinants would provide valuable insights for optimizing menopausal care and informing evidence-based clinical practice and policy.

## Conclusion

This nationally representative analysis revealed a significant increasing trend in the lifetime prevalence of MHT use among postmenopausal Korean women from 2005 to 2024, with overall prevalence rising from 13.8 to 17.5%. The most striking finding was the divergent age-specific pattern: dramatic increases among older women (≥70 years) coupled with declining prevalence among younger women aged 40–59 years. MHT use remained strongly associated with higher education and income levels, though these socioeconomic gradients narrowed somewhat in the recent period. These patterns may reflect evolving clinical practice and patient preferences in the post-WHI era in Korea, possibly influenced by accumulating evidence supporting individualized MHT use for symptomatic relief in appropriately selected women.

## Data Availability

Publicly available datasets were analyzed in this study. This data can be found here: https://knhanes.kdca.go.kr/knhanes/main.do.
